# A Novel, Five-Marker Alternative to CD16–CD14 Gating to Identify the Three Human Monocyte Subsets

**DOI:** 10.3389/fimmu.2019.01761

**Published:** 2019-07-26

**Authors:** Siew-Min Ong, Karen Teng, Evan Newell, Hao Chen, Jinmiao Chen, Thomas Loy, Tsin-Wen Yeo, Katja Fink, Siew-Cheng Wong

**Affiliations:** ^1^Singapore Immunology Network (SIgN), A*STAR Research Entities, Singapore, Singapore; ^2^School of Biological Sciences, Nanyang Technological University, Singapore, Singapore; ^3^National Centre for Infectious Diseases, Singapore, Singapore; ^4^Lee Kong Chian School of Medicine, Nanyang Technological University, Singapore, Singapore

**Keywords:** monocyte subsets, CD16, CD14, cytometry, dengue

## Abstract

Human primary monocytes are heterogeneous in terms of phenotype and function, but are sub-divided only based on CD16 and CD14 expression. CD16 expression distinguishes a subset of monocytes with highly pro-inflammatory properties from non-CD16 expressing “classical” monocytes. CD14 expression further subdivides the CD16^+^ monocytes into non-classical CD14^low^ and intermediate CD14^high^ subsets. This long-standing CD16–CD14 classification system, however, has limitations as CD14 is expressed in a continuum, leading to subjectivity in delineating the non-classical and intermediate subsets; in addition, CD16 expression is unstable, making identification of the subsets impossible after *in vitro* culture or during inflammatory conditions *in vivo*. Hence, we aimed to identify the three monocyte subsets using an alternative combination of markers. Additionally, we wanted to address whether the monocyte subset perturbations observed during infection is real or an artifact of differential CD16 and/or CD14 regulation. Using cytometry by time-of-flight (CyTOF), we studied the simultaneous expression of 34 monocyte markers on total monocytes, and derived a combination of five markers (CD33, CD86, CD64, HLA-DR, and CCR2), that could objectively delineate the three subsets. Using these markers, we could also distinguish CD16^+^ monocytes from CD16^−^ monocytes after *in vitro* stimulation. Finally, we found that the observed expansion of intermediate (CD14^high^) monocytes in dengue virus-infected patients was due to up-regulated CD16 expression on classical monocytes. With our new combination of markers, we can now identify monocyte subsets without CD16 and CD14, and accurately re-examine monocyte subset perturbations in diseases.

## Introduction

CD16^+^ human monocytes have gained much research interest due to their apparent expansion in various inflammatory diseases (1). These CD16^+^ monocytes consists of CD14^high^ and CD14^low^ populations (2), which expand independently of each other in different disease settings (3). Hence in 2010, the CD16^+^ subset is officially sub-divided into two subsets, intermediate (ITM; CD14^high^/CD16^+^) and non-classical (NC; CD14^low^/CD16^+^) (4), while the CD16^−^ monocytes form the classical (CL) subset. However, difficulties lie in distinguishing the ITM and NC subsets as CD14 is expressed in a continuum, and the flow cytometry plot profile for CD16 vs. CD14 varies between individuals (5). These led to a proposal for the “trapezoid” rather than “rectangular” gating strategy to discriminate between the ITM and NC subsets (6, 7). This subjective nature of gating leads to variation when identifying subsets (5), and potential discrepancies in experimental conclusions. To improve subset identification and purity, the use of additional gating steps with more markers such as CCR2, CD36, HLA-DR, and CD11c was suggested (8). The use of SLAN to distinguish ITM from NC monocytes was also proposed (9). However, these two methods still rely on the use of CD16 and CD14 in the initial steps of the gating, and the expression level of CD16 and CD14 changes rapidly *in vitro*, rendering the identification of subsets after *in vitro* stimulation impossible. There is thus a call for novel markers that are relatively stable *in vitro*, and are able to unequivocally distinguish the three subsets (10).

The three monocyte subsets exhibit unique roles at different stages of maturation. Monocytes emerge from the bone marrow as CL monocytes, and then differentiate into ITM and NC monocytes over a 12-day period (11–13). During this process, the monocytes undergo cellular senescence (14). Transcriptomic analyses have indicated that the three subsets have different functional specializations, especially during inflammation (15–17). As such, using only two markers, CD16 and CD14, to distinguish between these subsets has thus been representative, but simplistic.

A long-standing question regarding CD16^+^ subset expansion in various disease conditions, is whether this subset truly expands or if it is an artifact of a change in CD16 and/or CD14 expression on some monocytes, since the expression of CD16 and CD14 has been observed to be unstable *in vitro*. A real expansion of a subset necessitates that all cells in the expanded subset possess the phenotypic and functional characteristics of that particular subset. Conversely, differential CD16 and/or CD14 expression implicates that some cells in the expanded subset still carry features of the subset they originated from. Deciphering what leads to the observed expansion of a particular subset is crucial to further our understanding of the immune response to different diseases, but this cannot be achieved using the current CD16–CD14 classification system.

Here we used cytometry by time of flight (CyTOF) to identify a new combination of markers that can objectively delineate the three monocyte subsets, immediately *ex vivo* and after culturing *in vitro*. We then applied our marker combination to assess whether the expansion of a monocyte subset in acute dengue virus infection is real, or the result of differential CD16 and/or CD14 marker expression.

## Materials and Methods

### Patient Consent and Ethical Review

Human blood sample collection and all experimental procedures were approved by the Institutional Review Board, Singapore. Written informed consent was obtained from all participants in accordance with the Declaration of Helsinki. Healthy participants were recruited from volunteers at SIgN (IRB reference: 2017/2806). Apheresis cones were obtained from anonymous platelet donors (IRB reference: 2017–2512). Dengue participants were recruited from patients admitted to Tan Tock Seng Hospital who presented acute symptoms of dengue infection and were later confirmed by PCR for viral RNA. Recovered patients were followed up 1–2 weeks after discharge from the hospital (IRB reference: 2016/00982).

### CyTOF

Peripheral blood mononuclear cells (PBMCs) of eight healthy donors were obtained by Ficoll density gradient centrifugation and used for CyTOF. Cells were plated and stained in a U-bottom 96-well plate (BD Falcon, cat. no. 3077). First, cells were washed once with 200 μL of PBS and then stained with 100 μL of 200 μM cisplatin (Sigma-Aldrich, cat. no. 479306-1G) for 5 min on ice to exclude dead cell. Cells were washed twice with staining buffer (4% FBS, 2 mM EDTA, 0.05% Azide in 1× PBS) and stained with 50 μL of fluorophore-tagged antibodies for 30 min on ice. After two washes with staining buffer, cells were incubated in 50 μL of heavy-metal isotope–labeled surface Ab cocktail for 30 min on ice. Cells were washed twice with staining buffer then once with PBS before fixing with 200 μL 2% PFA (Electron Microscopy Sciences, cat. no. 15710) in PBS at 4°C overnight. Cells were then washed twice with 1x perm buffer (BioLegend, cat. no. 421002) and stained with 50 μL of metal isotope-labeled intracellular antibodies at room temperature. After 45 min, cells were washed once with perm buffer and then PBS before barcoding. Bromoacetamidobenzyl-EDTA (BABE)-linked metal barcodes were prepared by dissolving BABE (Dojindo, cat. no. B437) in 100 mM HEPES buffer (Gibco, cat. no. 15630) to a final concentration of 2 mM. Then, isotopically purified PdCl2 (Trace Sciences, Inc.) was added to BABE solution to 0.5 mM. Similarly, DOTA-maleimide (DM)-linked metal barcodes were prepared by dissolving DM (Macrocyclics, cat. no. B-272) in L buffer (MAXPAR, cat. no. PN00008) to a final concentration of 1 mM. Then, 50 mM of RhCl3 (Sigma) and isotopically purified LnCl3 (Trace Sciences, Inc.) was added to DM solution to 0.5 mM. A unique, dual combination of barcodes was chosen to stain each PBMC sample. Cells were incubated in 100 μL barcodes in PBS for 30 min on ice. Cells were then washed in perm buffer and incubated in staining buffer for 10 min on ice. Cells were then pelleted and resuspended in 100 μL of 250 nm iridium intercalator (MAXPAR, cat. no. 201192B) in 2% PFA/PBS at room temperature. After 20 min, cells were washed twice with staining buffer and twice with water before final resuspension in water at 0.5 × 10^6^ cells/mL prior to CyTOF acquisition. Cells were analyzed using a CyTOF mass cytometer (CyTOF 1, DVS Sciences). The data were exported in flow-cytometry file (FCS) format, and cells for each barcode were deconvolved by Boolean gating using FlowJo software (Tree Star, Ashland, USA). Data were analyzed using CyTOF kit (18) and FlowJo software (TreeStar).

### Flow Cytometry

PBMCs were stained with live/dead fixable dye (Invitrogen) for 30 min at room temperature, and labeled with the following antibodies for 20 min at 4°C: CD14 (#562335, BD Biosciences), CD16 (#302018, Biolegend), CD56 (#318344, Biolegend), CD33 (#562854, BD Biosciences), CD86 (#305412, Biolegend), CD64 (#IM1604U, Beckman Coulter), CCR2 (#FAB151P, R&D Systems), HLA-DR (#4333608, Thermo Fisher Scientific). Samples were acquired on a BD Fortessa flow cytometer (BD Biosciences) and analyzed using FlowJo software (TreeStar).

### Monocyte Sorting

PBMCs were depleted of granulocytes and lymphocytes using anti-CD15, anti-CD56, anti-CD3, and anti-CD19 microbeads (Miltenyi Biotec). The enriched monocyte fraction was labeled with anti-CD14, anti-CD16, and anti-CD56 for fluorescence-activated cell sorting (FACS) into the three monocyte subsets. CD14^−^CD56^+^ NK cells were excluded, as they also express CD16 (**Figure 2Cb**). The remaining cells were gated into CL (CD14^high^/CD16^−^), ITM (CD14^high^/CD16^+^), and NC (CD14^low^/CD16^+^) subsets (**Figure 2Cd**).

### Cell Culture

Monocytes were cultured in Iscove's Modified Dulbecco's Medium (IMDM; Hyclone) supplemented with 5% human serum (Innovative Research) and 1% penicillin/streptomycin (Invitrogen) in a humidified 37°C incubator with 5% CO_2_. For lipopolysaccharide (LPS) stimulation, 100 ng/ml LPS (*E. coli* serotype O111:B4) was added to the culture medium for the indicated duration.

### Comparing Conventional and New Gating Strategies

Sorted monocyte subsets were analyzed after 2 h *in vitro*, using the new gating strategy to calculate the percentage of each sorted subset that overlapped with its respective “New” subset gate. The average of two sorted samples was used for this analysis, and the calculation was based on 100 total monocytes. Sorting the 100 monocytes according to the conventional gating strategy resulted in 62.7 CL, 8.9 ITM, and 24.2 NC monocytes (Tables 2A,B); 4.2 cells did not fall into any of the three gates. We then assessed the proportions of sorted CL, ITM, and NC cells that overlapped with the “New” CL, ITM, and NC gates (Table 2A). Based on the percentages obtained on the flow cytometry plots (**Figure 4A**), the New CL gate contained 40.8 sorted CL monocytes, 0.9 sorted ITM monocytes, and 0.1 sorted NC monocytes. The New ITM gate contained 15.2 sorted CL monocytes, 4.5 sorted ITM monocytes, and 0.6 sorted NC monocytes. Finally, the New NC gate contained 0.2 sorted CL monocytes, 1.9 sorted ITM monocytes, and 19.9 sorted NC monocytes. The purity of each New subset gate was calculated by this formula: Purity of each New subset_X_ gate = (no. of sorted subset_X_ cells/total no. cells in New subset_X_ gate) × 100%, where subset_X_ is CL or ITM or NC. For example, Purity of New CL gate = (no. of sorted CL cells/total no. cells in New CL gate) × 100% = (40.8/[40.8 + 0.9 + 0.1]) × 100% = 97.6%. Likewise, the New ITM and New NC gates had a purity of 22.2 and 90.2%, respectively (Table 2A). Similarly, we assessed the proportions of sorted CL, ITM, and NC cells that overlapped with the “New” CL, ITM, and NC gates in stimulated condition (Table 2B), based on the percentages obtained on the flow cytometry plots (**Figure 4B**).

### Statistical Analyses

For comparisons between three groups (healthy, dengue, and recovered, **Figure 6**), one-way ANOVA was performed, with Tukey's Test to correct for multiple comparisons. *p < 0.05, ^**^p < 0.01, ^***^p < 0.001, ^****^p < 0.0001.

## Results

### CD16 Distinguishes a Subset of Monocytes With a Unique Phenotypic Profile

**Figure 1:**
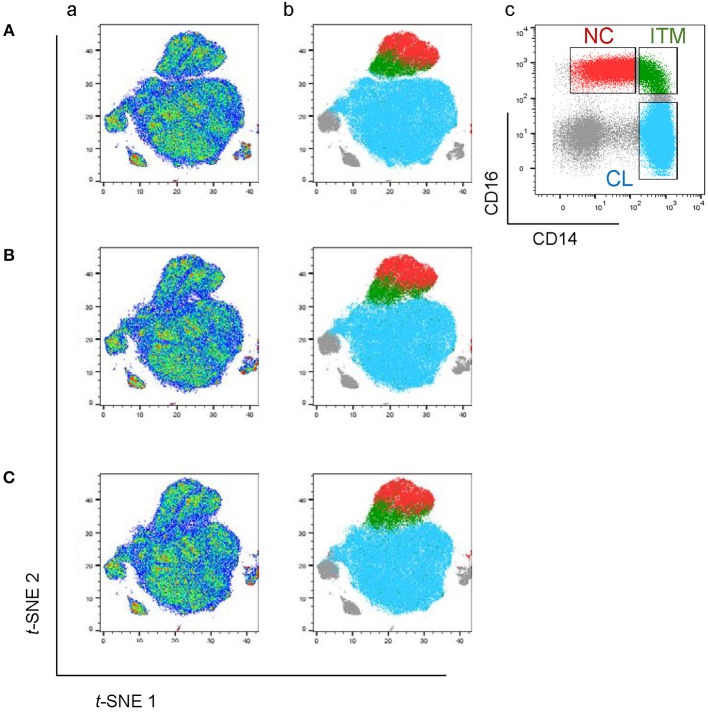
The CD16^+^ monocytes differ phenotypically from CD16^−^ monocytes. **(A)** *t*-SNE clustering of monocytes using all 34 markers (a) and with the three monocyte subsets overlaid (b). (c) Identification of the three subsets based on the conventional CD16–CD14 plot. **(B)** *t*-SNE clustering of monocytes without CD16. **(C)** *t*-SNE clustering of monocytes without CD16 and CD14.

We assembled a panel of 40 cell surface markers (6 markers for immune cell lineages and 34 monocyte markers) to label PBMCs isolated from eight healthy donors (Table 1). We then used t-distributed stochastic neighbor embedding (*t*-SNE) to visualize the expression of all 40 markers of all eight samples on a single plot (18). Cells that are similar in their surface marker-expression pattern are placed closely together on the *t*-SNE plot (19). As predicted, the main immune-cell populations clustered neatly, with the monocytes found in clusters #3 (CD16^+^) and #4 (CD16^−^) (Supplementary Figure 1). To focus on monocytes, we gated out T cells (CD3), B cells (CD19), natural killer (NK) cells (CD56, CD57, and CD7), and neutrophils (CD66b). We then re-analyzed the remaining cells, which mainly consisted of monocytes and dendritic cells (DCs), using the 34 monocyte markers. The resulting *t*-SNE plot showed two main tightly-connected clusters of cells (monocytes), and a few small, distant clusters (Figure 1Aa).

**Table 1:** Markers to phenotype total monocytes by CyTOF.

**Group**	**Marker**	**Isotope Tag**
Lineage markers	CD45	La-139
	CD3	Pm-147
	CD19	Gd-156
	CD7	Nd-144
	CD57	In-113
	CD66b	Sm-149
Monocyte subset identification	CD14	Cd-112/114
	CD16	Sm-154
Fcγ receptors	CD32	Nd-150
	CD64	Eu-151
Myeloid markers	CD33	Tb-159
	CD68	Gd-155
Receptors for growth factors	CD115	Er-166
	CD114	Dy-164
Scavenging receptors	CD36	Nd-145
	CD163	Tm-169
Endocytic receptors	CLEC4D	Gd-160
	CLEC5A	Yb-171
Adhesion molecules	CD11b	Lu-176
	CD54	Lu-175
	CD62L	Nd-143
	Siglec10	Gd-157
Antigen presentation	HLA-DR	Nd-142
	CD86	Eu-153
	CD43	Nd-146
Chemokine receptors	CCR1	Dy-162
	CCR2	Er-168
	CCR5	Yb-173
	CXCR1	Ho-165
	CX_3_CR1	Yb-174
DC markers	CD1c	Dy-163
	CD141	Er-170
	CD123	Dy-161
	FcεR1α	Sm-152
Others	CD9	Gd-158
	CD99	Pr-141
	SLAN	Er-167
	VSTM1	Yb-172
	CD15	In-155
	CD56	Nd-148
	DNA	Ir-191/193
	Cisplatin live/lead	195
Barcode		Rh-103
		Pd-104
		Pd-105
		Pd-106
		Pd-108
		Pd-110

To locate the three monocyte subsets on the *t*-SNE plot, we gated based on the conventional CD16–CD14 two-dimensional plot (Figure 1Ac), and then overlaid this onto the *t*-SNE plot (Figure 1Ab). The two CD16^+^ subsets, ITM (green) and NC (red), clustered together and away from the CL subset (blue). This implies that the two CD16^+^ subsets are very similar to each other, and less similar to CD16^−^ monocytes, based on the expression of the 34 monocyte markers. The un-gated cells (gray) were cells which did not express CD16 or CD14, consisting mainly of DCs.

We next assessed how accurately monocyte subsets can be distinguished using only CD16. Excluding CD16 produced a *t*-SNE plot that looked similar to the plot obtained previously, except that now the ITM subset was less distinctly separated from the CL subset (Figure 1B). This finding strongly supports that CD16^+^ monocytes exhibit a unique phenotypic profile, but CD16 is not essential to set CD16^+^ monocytes apart from CD16^−^ monocytes. We then performed a further analysis to determine whether CD14, after excluding CD16, is also dispensable for monocyte subset differentiation. The *t*-SNE plot generated by excluding both CD16 and CD14 (Figure 1C) was very similar to the previous plot (Figure 1B). These results imply that all three subsets differ in monocyte marker expression beyond CD16 and CD14, hence alternative markers may be able to delineate the three subsets.

### A Novel Combination of Five Markers Identifies Three Monocyte Subsets

**Figure 2:**
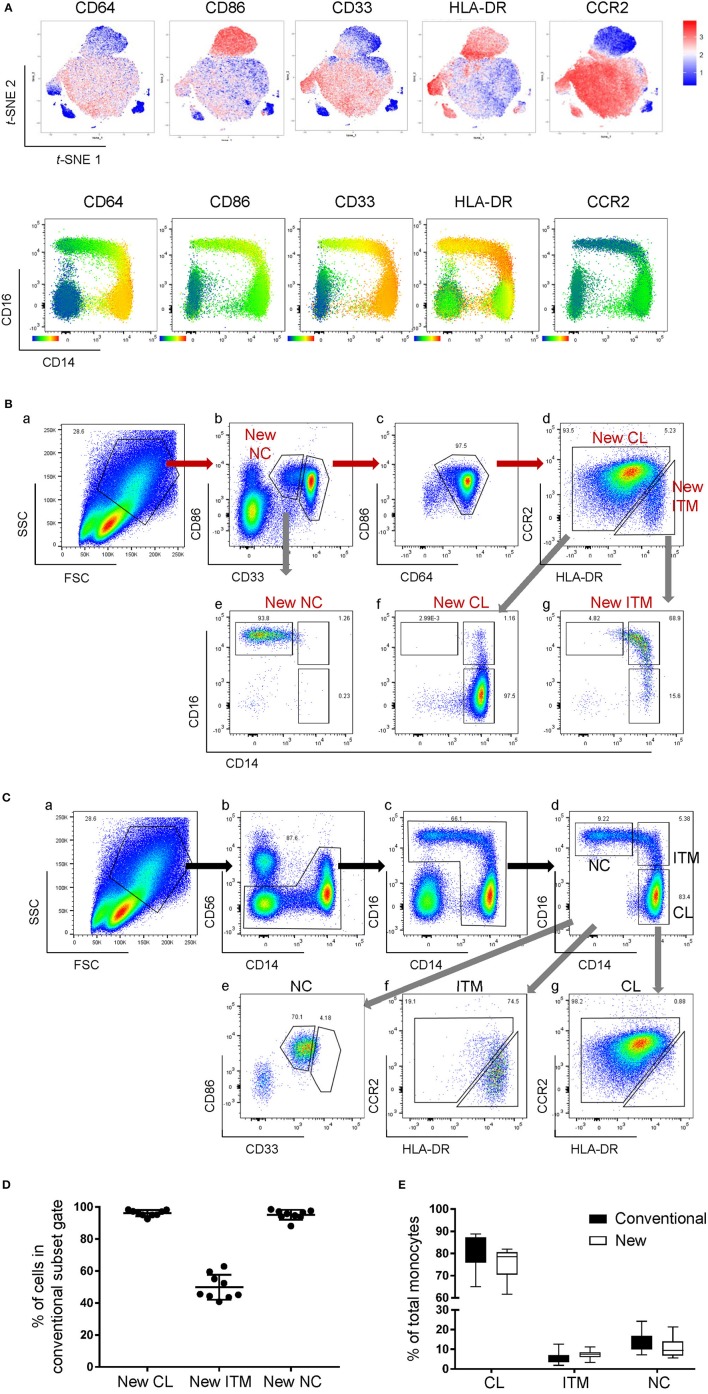
A new marker combination objectively identifies monocyte subsets. **(A)** Expression heatmaps of the five selected markers on total monocytes on *t*-SNE plots using data from CyTOF (top panel) and on CD16-CD14 plots using data from flow cytometry (bottom panel). **(B)** The new gating strategy for the three subsets using the five selected markers. **(C)** Analyzing the conventionally-gated subsets with the new gating strategy. **(D)** Purity of the New subset gates. Data represent the means ± SD. **(E)** Proportion of each subset as a percentage of total monocytes using the two different gating strategies. Data represent the means ± SD.

We next studied the expression of the remaining 32 monocyte markers on the *t*-SNE plots (Supplementary Figure 2) to find replacements for CD16 and CD14 to identify the subsets. The criteria for selection of our marker combination is as follow: Firstly, the markers must be able to distinguish the CD16^+^ from the CD16^−^ monocytes. Secondly, the markers must be able to distinguish NC subset from the ITM subset. Thirdly, the markers must be selective for monocytes. Finally, the marker combination should consist of the least number of markers necessary to achieve the above three aims, in order to allow researchers to study other markers of interest on monocytes and save cost on antibodies. To distinguish the CD16^+^ from the CD16^−^ monocytes, the following markers were shortlisted: CD64, CD115, HLA-DR, CD86, CCR2, CD15, CD99, and CX_3_CR1. To distinguish NC from ITM monocytes, the following markers were shortlisted: CD33, CD36, CD11b, and SLAN. In addition, CD64, CD33, and CD86 are also selective for monocytes. As the new marker combination must be reproducible on a widely-available platform, we validated these markers by flow cytometry. We labeled PBMCs with the shortlisted markers, together with the three conventional markers (CD14, CD16, and CD56) used to identify the subsets, and studied the expression of the shortlisted markers on a conventional CD16–CD14 plot (Supplementary Figure 3A). The markers CD115 and CD15 were eliminated due to low overall expression on monocytes, while CD99 was eliminated due to its high expression on non-monocytes (the CD14^−^/CD16^−^ cells; Supplementary Figure 3B). SLAN was present only on a small proportion of the NC subset and its expression varied widely between samples, and was thus eliminated too (Supplementary Figure 3C). We then tested different combinations of the remaining markers, CD64, HLA-DR, CD86, CX_3_CR1, CCR2, CD33, CD36, and CD11b, to find the best combination with the least number of markers that could identify the three monocyte subsets while excluding non-monocytes. CX_3_CR1, CD36, and CD11b were eliminated as they were expressed in a continuum on monocytes such that they could not delineate the subsets. Finally, we selected five markers (Figure 2A): CD64, CD86, and CD33 to exclude non-monocytes, with CD33 doubling up to distinguish between ITM and NC monocytes, and CCR2 and HLA-DR to distinguish CL and ITM monocytes. With these five markers, we developed a new gating strategy for the three monocyte subsets. After gating on single cells and live cells, we gated on the monocyte population (Figure 2Ba), deliberately including a small portion of the lymphocyte population as NC monocytes are smaller in size and tend to overlap with the lymphocytes. From this monocyte population, the CD33^+^/CD86^+^ cells consisted of monocytes (Figure 2Bb). Of these cells, the CD33^low^ cells consisted of NC monocytes, which we termed “New NC” monocytes. Back-gating the New NC monocytes onto a CD16–CD14 plot confirmed that this population mainly consisted of conventionally-defined NC monocytes (Figure 2Be). From the CD33^high^ cells (Figure 2Bb), we gated out CD64^low^ cells (Figure 2Bc), which mainly consisted of DCs. From the CD64^high^ cells, the CCR2^high^ cells mainly consisted of CL monocytes, which we termed “New CL” monocytes, while the HLA-DR^high^ cells mainly consisted of ITM monocytes, or “New ITM” monocytes (Figure 2Bd). Back-gating these two populations onto a CD16–CD14 plot confirmed that the New CL and New ITM gates mainly consisted of the conventionally-defined CL and ITM monocytes, respectively (Figures 2Bf,g). For comparison, we studied the conventionally-gated subsets with our new gating strategy (Figure 2C). From the monocyte population, we first gated out NK cells (CD56^+^ CD14^−^ cells; Figure 2Cb), which would otherwise fall into the NC gate. Next, we gated out CD14^−^ CD16^−^ cells, which consisted of lymphocytes and DCs (Figure 2Cc). Finally, we gated for the three subsets according to the conventional CD16–CD14 gating (Figure 2Cd). Back-gating them onto our New subset gates confirmed that the New subset gates could identify the conventionally-gated subsets (Figures 2Ce–g).

To compare the new and conventional gating methods, we analyzed the percentage of cells in the New subset gates that overlapped with their respective conventional subset gates. More than 95% of cells in the New CL and New NC gates overlapped with their respective conventional gates, while 50% of New ITM overlapped with the conventional ITM gate (Figure 2D), showing that the new gating strategy works well for CL and NC subsets. Next, we compared the proportion of the three subsets as a percentage of all monocytes between the new and conventional gating strategies (Figure 2E): the new gating strategy yielded a slightly lower percentage of CL [75.3% (new) vs. 80.6% (conventional)] and NC subsets [10.8% (new) vs. 13.6% (conventional)], and a slightly higher percentage of ITM [7.5% (new) vs. 5.5% (conventional)]. We thus propose that CD86, CD33, CD64, CCR2, and HLA-DR can be used to identify the three monocyte subsets. Furthermore, CD33 and CD86 (Figure 2Bb) can more objectively separate NC from ITM cells, compared to using CD14 in the conventional CD16–CD14 plot.

### The Novel Combination of Five Markers Separates CD16^+^ and CD16^−^ Monocytes After Stimulation *in vitro*

**Figure 3:**
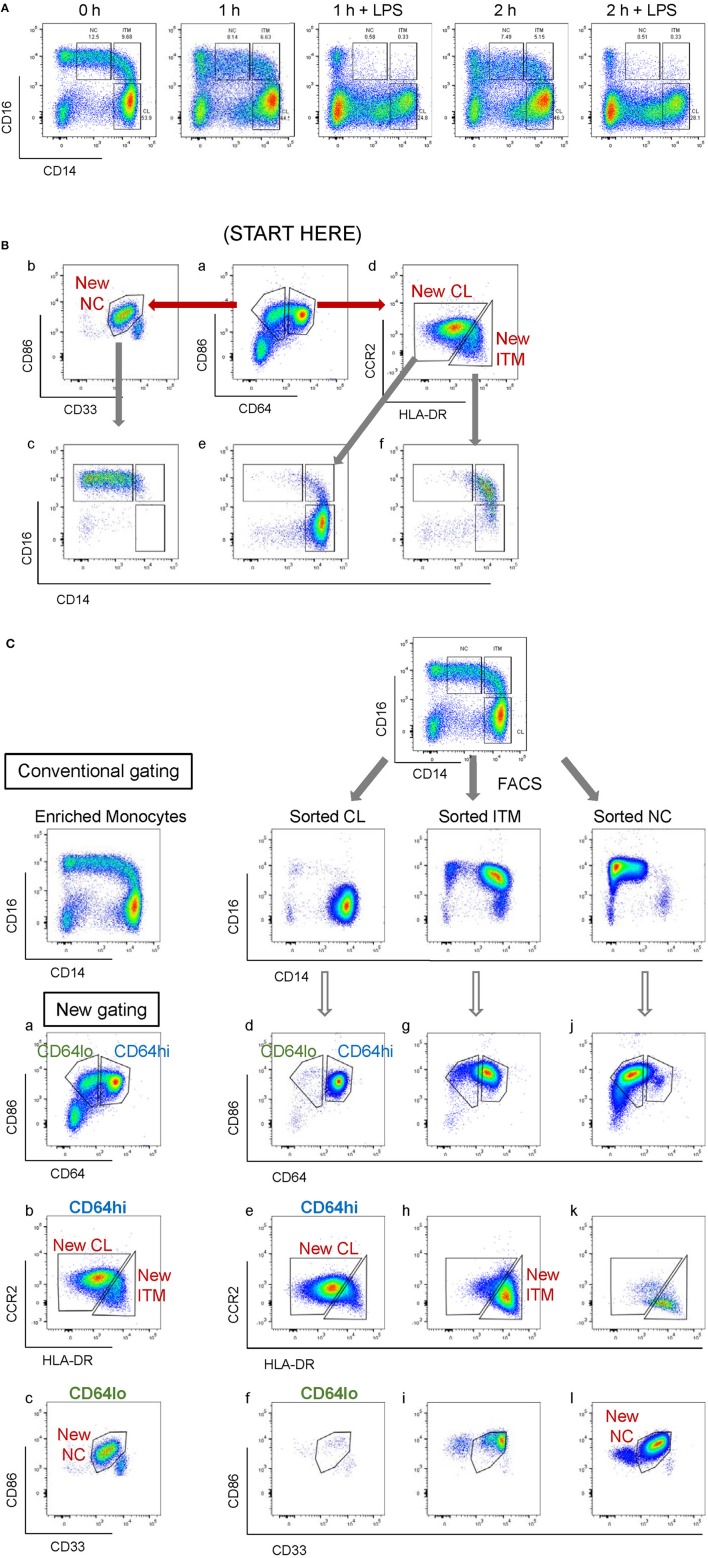
Application of the new marker combination on monocytes *in vitro*. **(A)** Instability of CD16 and CD14 expression *in vitro*. **(B)** The modified gating sequence for cultured cells. **(C)** Analysis of sorted monocytes subsets for modulation of new markers *in vitro*.

**Figure 4:**
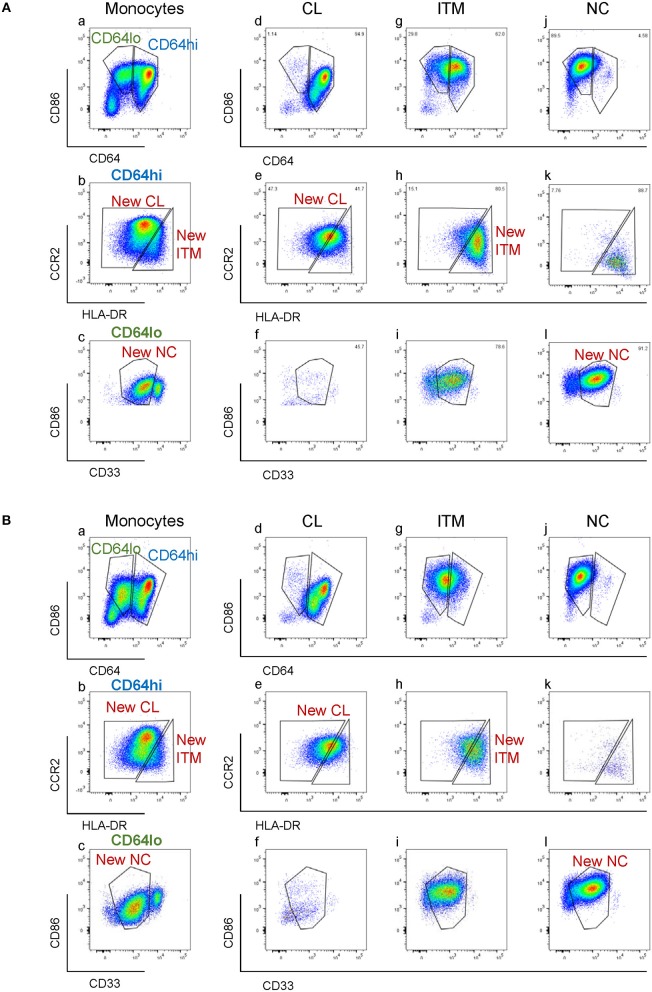
Modulation of new subset markers *in vitro*. **(A)** Analysis of marker modulation on sorted subsets after 2 h culture without stimulation. **(B)** Analysis of marker modulation on sorted subsets after 2 h with LPS stimulation.

CD16 and CD14 expression on monocytes decreases rapidly *in vitro*. After 2 h, CD16 expression was lost, while a CD14^low^ population appeared, and the CD14^−^ population increased (Figure 3A). These changes were more pronounced with LPS stimulation, in both PBMC (Figure 3A) and whole blood (Supplementary Figure 4). Consequently, gating monocyte subsets using CD16 and CD14 after culture or stimulation *in vitro* becomes impossible. Thus, any new marker system proposed to identify the subsets should maintain stable expression *in vitro*. We assessed the stability of our five markers on monocytes cultured for up to 2 h *in vitro*. Expression of the five markers varied, but to a lesser extent than CD16 and CD14. As CD64 expression was more stable than CD33 (Supplementary Figure 5), we modified our gating sequence for cultured cells, to obtain the same New subsets (Figure 3B). From the monocyte population, we first gated on CD64^+^ and CD86^+^ cells (Figure 3Ba), which mainly consisted of monocytes. From the CD64^low^ cells, we gated on CD33^low^ cells to obtain the New NC subset (Figure 3Bb). Back-gating this New NC subset onto the CD16–CD14 plot confirmed that this gate mainly consisted of NC cells (Figure 3Bc). From the CD64^high^ cells (Figure 3Ba), the CCR2^high^ cells made the New CL subset, and the HLA-DR^high^ cells made the New ITM subset (Figure 3Bd). Back-gating these two subsets onto the CD16–CD14 plot confirmed their identities (Figures 3Be,f). In parallel, we sorted monocytes into three subsets by conventional CD16–CD14 gating (Figure 3C). Using the monocytes as a reference to gate for the three subsets (Figures 3Ca–c), we applied these New subset gates to the sorted subsets (Figures 3Cd–l) to assess where the sorted subsets sit in their new gates. Using this gating strategy on uncultured cells as a reference, we performed the same analysis on monocytes and sorted subsets cultured for 2 h, to assess the extent to which the sorted subsets can be identified by the New subset gates after culture. Figures 4Aa–c show how we would have gated on monocytes after culture with our new gating strategy. These same gates were then applied to the sorted subsets (Figures 4Ad–l), which showed how much the three subsets have drifted from their new gates due to changes in the expression of the five markers. After 2 h of culture, a substantial proportion (39.6 ± 21.4 %) of sorted CL monocytes have moved into the New ITM gate (Figure 4Ae), which would have “contaminated” the New ITM gate with CL cells. Likewise, for the sorted ITM cells, 10.5 ± 1.7% have moved into New CL gate (Figure 4Ah) and 20.9 ±3.8% into the New NC gate (Figure 4Ai). For the sorted NC monocytes, 2.6 ± 2% have moved into the New ITM gate (Figure 4Ak). To quantify the extent to which this new gating strategy allows us to identify the subsets after culture, we computed the percentage of the sorted subsets that remained in their respective new gates. To do so, we had to take into account that a sample of total monocytes contained different numbers of cells from each subset, which would affect the extent of “contamination” of the New gates. For instance, a drift of 10% of the sorted ITM cells into the New CL gate would have effects far less than a drift of 10% of the sorted CL cells into the New ITM gate due to the larger number (~7 times) of CL cells than ITM cells in a total monocyte sample. Hence, we based the calculation on a starting sample of 100 total monocytes (see section Materials and Methods), which would give an average of 62.7 CL, 8.9 ITM, and 24.4 NC monocytes. The net result with no stimulation was as follows: the New CL gate contained 97.6% cells from the sorted CL subset, the New ITM subset contained 22.2% cells from the sorted ITM subset, and the New NC subset contained 90.2% cells from the sorted NC subset (Table 2A). With LPS stimulation (Figure 4B), the New CL gate contained 97.3% cells from the sorted CL subset, the New ITM subset contained 12.1% cells from the sorted ITM subset, and the New NC subset contained 77.7% cells from the sorted NC subset (Table 2B).

**Table 2:** Purity of three New subset gates after 2 h *in vitro* unstimulated **(A)** and stimulated **(B)**.

	**No. of cells**			**% Purity of New gate**
	**CL (62.7)**	**ITM (8.9)**	**NC (24.2)**	
**(A) 2 h, unstimulated**				
New CL	**40.8**	0.9	0.1	97.6
New ITM	15.2	**4.5**	0.6	22.2
New NC	0.2	1.9	**19.9**	90.2
Undetermined	6.4	1.5	3.6	–
**(B) 2 h, with LPS stimulation**				
New CL	**41.8**	1.1	0.1	97.3
New ITM	11.4	**1.6**	0.1	12.1
New NC	1.7	4.7	**22.3**	77.7
Undetermined	7.9	1.5	1.7	–

*Bold values indicate the number of sorted cells which remained in their respective New subset gate, which is used for calculating % purity of New gate*.

These data show that the new gating strategy can identify CL monocytes to a good degree (~97% purity) and NC monocytes to a decent degree (77–90%), but not ITM monocytes (12–22%), after culturing. However, we saw an opportunity to use the New NC gate for identifying total CD16^+^ monocytes after culture, because the New NC gate consisted mainly of ITM and NC cells, making up a total of 99.1% after 2 h without stimulation (Table 3A), and 94.1% with LPS stimulation (Table 3B). Hence, we propose that the new gating strategy can be used to distinguish CD16^+^ and CD16^−^ subsets after *in vitro* culture, even with LPS stimulation.

**Table 3:** Purity of two New subset gates after 2 h *in vitro* unstimulated **(A)** and stimulated **(B)**.

	**No. of cells**		**% Purity of New gate**
	**CL**	**CD16^**+**^ (ITM and NC)**	
**(A) 2 h, unstimulated**			
New CL	**40.8**	1.0	97.6
New ITM	–	–	–
New NC	0.2	**21.8**	99.1
**(B) 2 h, with LPS stimulation**			
New CL	**41.8**	1.2	97.2
New ITM	–	–	–
New NC	1.7	**27.0**	94.1

*Bold values indicate the number of sorted cells which remained in their respective New subset gate, which is used for calculating % purity of New gate*.

### The Novel Combination of Five Markers Eliminates Non-monocytes From NC Gate

**Figure 5:**
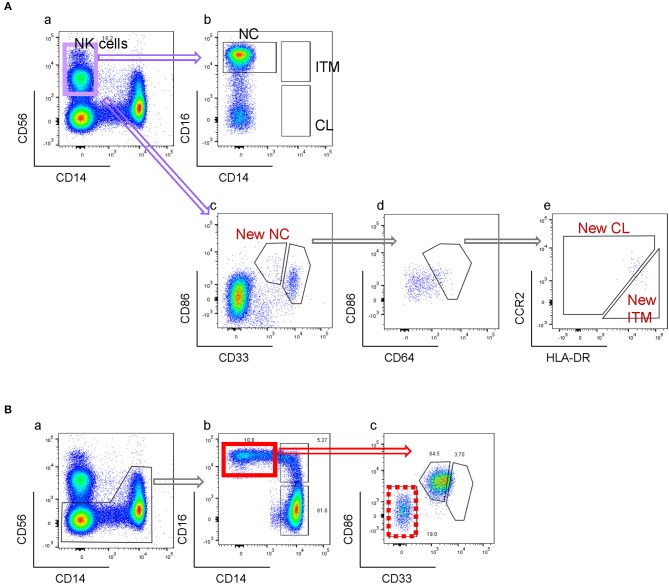
New gating strategy eliminates non-monocytes. **(A)** Elimination of NK cells. **(B)** Elimination of other CD16^+^ non-monocytes.

By the conventional CD16–CD14 gating, non-monocytes can contaminate the NC gate due to its CD14^low^ property. NK cells, which are CD14^−^ and CD16^+^, form the largest contaminating cell population (Figures 5Aa,b). Consequently, an NK marker such as CD56 was used to eliminate them from the NC gate. However, as NK cells do not express CD33 and CD86, our new marker combination excludes NK cells without requiring an additional NK marker (Figure 5Ac). The rare NK sub-population that falls into the CD33^high^ gate is removed in the subsequent CD64-gating step (Figures 5Ad,e). Other than NK cells, we found that the conventional NC gate still contained other non-monocytes, likely γδ-T cells or DCs, which were also excluded with the markers CD33 and CD86 (Figure 5B). Hence our new combination of markers eliminates non-monocytes without requiring additional markers for different contaminating cell types.

### The Five-Marker Gating Clarifies Monocyte Subset Perturbations During Dengue Infection

**Figure 6:**
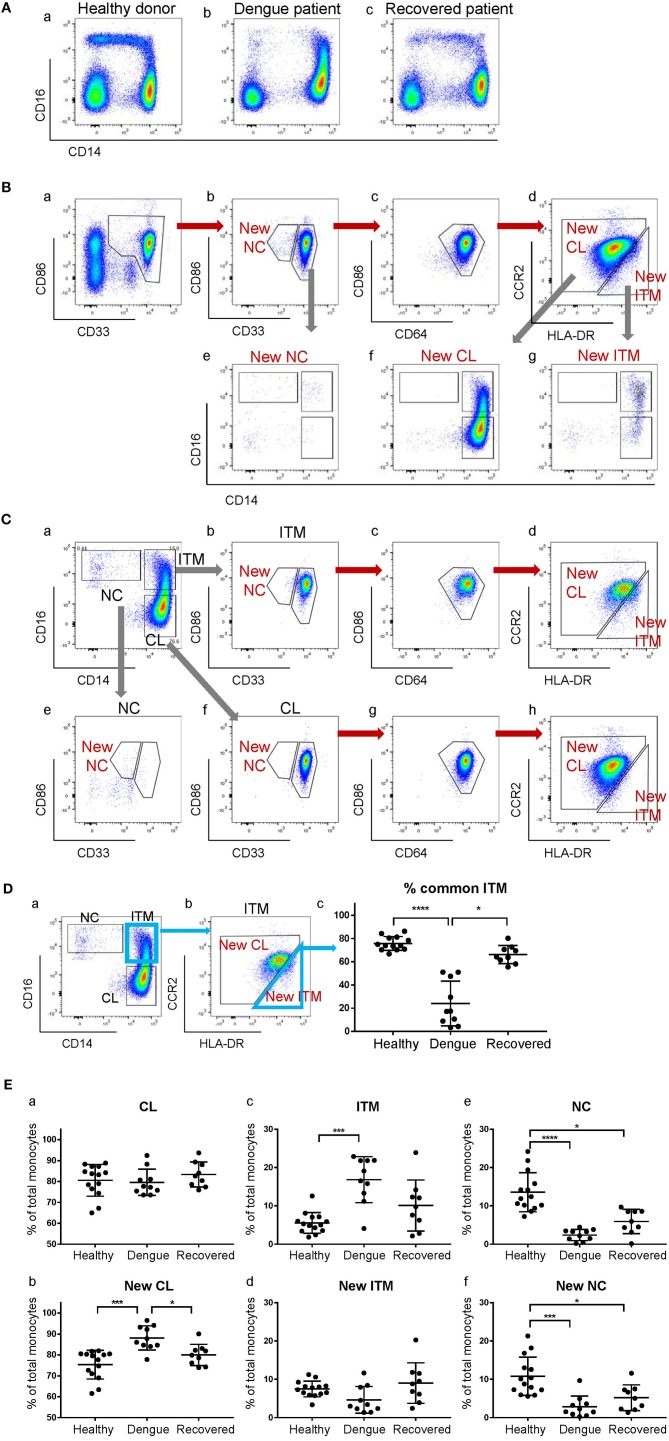
The expanded ITM subset in dengue patients originates from the CL subset. **(A)** CD16–CD14 profile of monocytes in a representative healthy donor, a dengue patient, and a recovered patient. **(B)** Analysis of monocytes from a dengue patient using the new gating strategy. **(C)** Analysis of conventionally-gated subsets with the new gating strategy. **(D)** Percentage (%) common ITM subset in healthy donors, dengue patients and recovered patients. Data represent the means ± SD. **(E)** Proportion of each subset as a percentage of total monocytes using the conventional (a,c,e) and new gating strategies (b,d,f). Data represent the means ± SD.

The CD16^+^ monocyte population (ITM or NC, or both) is reported to expand in numerous inflammatory conditions (3, 20). Here, we observed an expansion of the ITM subset, and a loss of the NC subset in patients infected with dengue virus (Figure 6Ab). Upon recovery, the ITM subset reduced and the NC subset re-appeared (Figure 6Ac). It is possible, however, that this ITM subset expansion and NC subset reduction is an artifact of CD16 up-regulation by some CL monocytes, or CD14 up-regulation by NC monocytes. To resolve this issue, we used our new strategy to analyse monocytes from dengue-infected patients (Figure 6B). We observed a loss of the New NC subset (Figure 6Bb), just like in the conventional gating, implying that the NC subset is truly reduced during dengue virus infection. However, we did not observe an expansion of the New ITM subset (Figure 6Bd). Instead, back-gating the New CL subset onto the conventional CD16–CD14 plot showed that the New CL subset spread over the conventional CL and ITM subsets (Figure 6Bf), suggesting that the expanded ITM subset resulted from CD16 up-regulation by CL monocytes and is not a true expansion of the ITM subset. To confirm this observation, we analyzed the conventionally-gated subsets with our new gating strategy (Figure 6C). Indeed, we saw that the ITM cells originated from the New CL gate (Figure 6Cd).

To quantify how much of the expanded ITM subset would also be identified as New ITM cells by the new gating strategy, we analyzed the percentage of the cells in the conventional ITM gate (Figure 6Da) that overlapped with the New ITM gate (Figure 6Db, blue triangle); we termed these cells “% common ITM” (Figure 6Dc). Healthy donors had a good 76% common ITM, while dengue patients had a significantly lower 24%. Upon recovery, the % common ITM increased significantly to 66%, a level similar to that of healthy donors. These observations imply that the upregulated ITM population reported in dengue patients may not be a true expansion of the ITM subset as previously reported, but mainly derives from an up-regulation of CD16 expression on a sub-population of CL monocytes.

Finally, we studied the proportion of the subsets as a percentage of total monocytes in healthy donors, dengue-infected and recovered patients (Figure 6E). By the conventional subset gating strategy, we found no differences in the CL subset between the three groups (Figure 6Ea). By the new gating strategy, however, the New CL subset was found to have significantly increased from 75 to 88% in dengue infection, and returned to near healthy levels (80%) upon recovery (Figure 6Eb). The ITM subset, according to conventional gating, significantly increased from 5.5 to 17% in dengue infection compared to healthy controls, and dropped to 10% upon recovery. Conversely, the New ITM subset showed a drop (7.5 to 4.6%) in dengue infection and an increase to 9.0% upon recovery (Figures 6Ec,d). Finally, the conventional NC subset significantly decreased from 14 to 2.4% in dengue infection and increased slightly to 6% upon recovery (Figure 6Ee). A similar trend was observed with the New NC subset, where it significantly dropped from 11 to 2.8% in dengue infection, and increased to 5.2% upon recovery (Figure 6Ef). In summary, the new gating strategy showed that the CL subset, rather than the ITM subset, expands during dengue infection. Additionally, the new gating strategy confirmed that the NC subset reduces during dengue infection.

## Discussion

Monocytes exhibit differential expression of a myriad of markers other than CD16 and CD14 (Supplementary Figure 2) (16, 17, 20, 21). Using only CD16 and CD14 for monocyte classification into three subsets is thus simplistic. Here, we studied the simultaneous expression of 34 markers on monocytes by CyTOF, and visualized the multi-dimensional expression on a single plot using *t*-SNE, where no manual gating is involved. We found that CD16^+^ monocytes, i.e., ITM and NC together, clustered together and away from the CD16^−^ monocytes. Excluding CD16 and CD14 in the analysis had little effect on the *t*-SNE map (Figure 1), implying that the difference in the 34-marker phenotypic profiles between the monocyte subsets was not heavily dependent on CD16 and CD14 expression. Based on the *t*-SNE map of the 32 markers on monocytes, we verified that using CD16 and CD14 to classify the monocyte into subsets is accurate and representative. We also observed that the ITM subset is phenotypically more similar to the NC than the CL subset, consistent with our previous transcriptomic study (16).

As monocyte subset clustering by *t*-SNE did not depend on CD16 and CD14 expression, we supposed that the subsets can be identified using other markers. The motivation is to identify the three subsets in a more objective way. One group proposed using SLAN to replace CD14 to distinguish ITM from NC monocytes, as SLAN is not expressed in a continuum by the CD16^+^ monocytes (9). The use of SLAN was substantiated by transcriptomic analyses of SLAN^+^ and SLAN^−^ subsets which revealed an ubiquitin signature not observed in CD14^low^ and CD14^high^ subsets. Furthermore, patients with sarcoidosis and MCSF-R mutation displayed perturbations in subset percentages only with the SLAN-based classification, signifying its biological relevance. Another group proposed the use of four additional markers CCR2, CD36, HLA-DR, and CD11c, to improve subset identification and purity (8). However, these two methods still rely on CD16 and CD14 before employing the new markers to identify the subsets.

We, instead, aimed to find a novel combination of markers to replace CD14 and CD16. From the 32 markers, we selected CD64, CD86, CD33, HLA-DR, and CCR2 (Figure 2A). We could objectively gate out the three subsets and achieve >95% purity for the New CL and New NC gates (Figure 2D). Unfortunately, the purity of the New ITM was only ~50%, which could be due to the high heterogeneity of the ITM subset, as reported in a single-cell RNA sequencing study, where the ITM monocytes were distributed among four monocyte clusters (22). It would be interesting to perform single-cell RNA sequencing on our New ITM subset to see if it is less heterogeneous than the conventional ITM subset. Importantly, we showed that our new gating strategy produces comparable subset percentages to conventional gating (Figure 2E), lending credibility to our new strategy.

Another rationale for replacing CD16 and CD14 markers is the instability of the two markers *in vitro*. Some groups have proposed HLA-DR as a replacement for CD16 under culture conditions when CD14 expression remained stable (23–25). However, we and others have found the expression of both CD16 and CD14 to drop rapidly in culture (24, 26, 27), especially when the monocytes are stimulated with LPS (Figure 3A and Supplementary Figure 4), rendering the gating for subsets impossible. Here, the expression of the five new markers was also modulated during culture (Figure 4). Notably, CD64 was more stable than CD33 during culture (Supplementary Figure 5), which inspired us to change the sequence of gating for cultured cells (Figure 3B). Compared to cells that have not been cultured, the gating for subsets was less objective, and the ITM and NC subsets could not be separated. Nonetheless, the five markers allowed us to separate CD16^+^ and CD16^−^ subsets after culturing *in vitro* (Figure 4 and Tables 2, 3). While this modified gating strategy works to identify CD16^+^ and CD16^−^ subsets after stimulation with LPS, it would be essential to check its suitability in culture conditions with other stimuli.

One limitation of the conventional gating method is the contamination of the NC gate by NK cells (20, 28), which have low CD14 and high CD16 expression (Figures 5Aa,b). In order to avoid the inclusion of NK cells in the NC subset gate, one way is to exclude CD14-very low-to-negative cells (5, 29, 30), which can result in up to 50% loss of the NC population. Another way is to exclude the NK cells using an NK marker before gating for the three subsets (Figures 2C, 5B). However, our new marker combination revealed that despite excluding NK cells, the NC gate still contained a substantial population of contaminating cells which were CD33^−^CD86^−^ (Figures 5Bb,c), likely to be γδ-T cells or DCs. Hence our new marker combination has the added advantage of excluding non-monocytes, more than just NK cells, from the NC gate while identifying the three monocyte subsets, without the need of additional markers.

The CD16^+^ subset reportedly expands in various infections (3, 20, 31, 32), but the role and cause of this expansion remains unclear. We questioned whether ITM and/or NC subset expansion in dengue virus infection was merely due to a modulation in CD14 and/or CD16 expression on some monocytes, or a true expansion of the subset(s). We profiled monocytes from infected patients by conventional gating and found that the ITM subset expanded while the NC subset reduced, in line with previous reports (3, 32). However, using our new gating strategy, we saw that most of the expanded ITM subset actually originated from the New CL gate, implying that the expanded ITM subset resulted from a CD16 up-regulation on some CL monocytes. No monocytes from the expanded ITM subset came from the New NC gate, indicating that the expansion did not result from CD14 up-regulation on NC monocytes (Figure 6). This observation is consistent with the current knowledge that monocytes undergo maturation from CL to ITM and NC subsets (13, 14, 25). We also saw very few cells in the New NC gate, in agreement with conventional gating. Interestingly, contrary to conventional gating, we saw an expansion of the New CL subset and a reduction of the New ITM subset. The reduction in New ITM was unlikely to be due to an up-regulation of CCR2 by the ITM monocytes, as CCR2 expression is known to be down-regulated with maturation of monocytes from CL to ITM to NC subsets (16, 33). With the expanded subset identified to be classical rather than intermediate, we propose the following: monocytogenesis in the bone marrow is induced during dengue infection; the increased numbers of monocytes enter the circulation as classical monocytes, with a proportion of them up-regulating CD16 expression in response to the infection. Majority of these cells migrate into tissues to repair infection-mediated damage, or die in circulation, leaving few cells to mature into non-classical monocytes in the circulation. One way to confirm that the expanded ITM subset originates from the CL subset would be to perform single-cell RNA sequencing on the monocytes from dengue patients. The origin of the expanded subset in disease conditions is a key question to pursue further evidence for, because if the expanded subset remains phenotypically similar to the subset it originates from, it would have very different functions from the subset it is perceived to be, and could change our understanding of the role of monocyte subsets in dengue pathogenesis.

In summary, we have developed a novel combination of five markers to objectively identify the three monocyte subsets. These markers can separate CD16^+^ and CD16^−^ monocytes after stimulation *in vitro* and can eliminate contaminating cells to obtain a relatively pure population of NC monocytes. Notably, our new markers may identify different subsets to be expanded or reduced during infections, compared to the conventional CD16–CD14 gating system.

## Data Availability

All datasets generated for this study are included in the manuscript and/or the Supplementary Files.

## Ethics Statement

Human blood sample collection and all experimental procedures were approved by the Institutional Review Board, Singapore. Written informed consent was obtained from all participants in accordance with the Declaration of Helsinki. Healthy participants were recruited from volunteers at SIgN (IRB reference: 2017/2806). Apheresis cones were obtained from anonymous platelet donors (IRB reference: 2017–2512). Dengue participants were recruited from patients admitted to Tan Tock Seng Hospital who presented acute symptoms of dengue infection and were later confirmed by PCR for viral RNA. Recovered patients were followed up 1–2 weeks after discharge from the hospital (IRB reference: 2016/00982).

## Author Contributions

S-MO designed and performed the experiments, analyzed data, and wrote the manuscript. KT, HC, and TL performed experiments. EN and JC advised on the planning of CyTOF experiments and data analysis. T-WY and KF are the research PI for the clinical study/IRB. S-CW supervised the study and edited the manuscript.

### Conflict of Interest Statement

The authors declare that the research was conducted in the absence of any commercial or financial relationships that could be construed as a potential conflict of interest.
