# Supplementary material for: A Novel, Five-Marker Alternative to CD16–CD14 Gating to Identify the Three Human Monocyte Subsets
Source: Front Immunol. 2019 Jul 26;10:1761. doi: 10.3389/fimmu.2019.01761 (PMC6676221; doi:10.3389/fimmu.2019.01761)
Supplement: Supplementary file 1 [file Data_Sheet_1.PDF]

Supplementary Figure S1

A

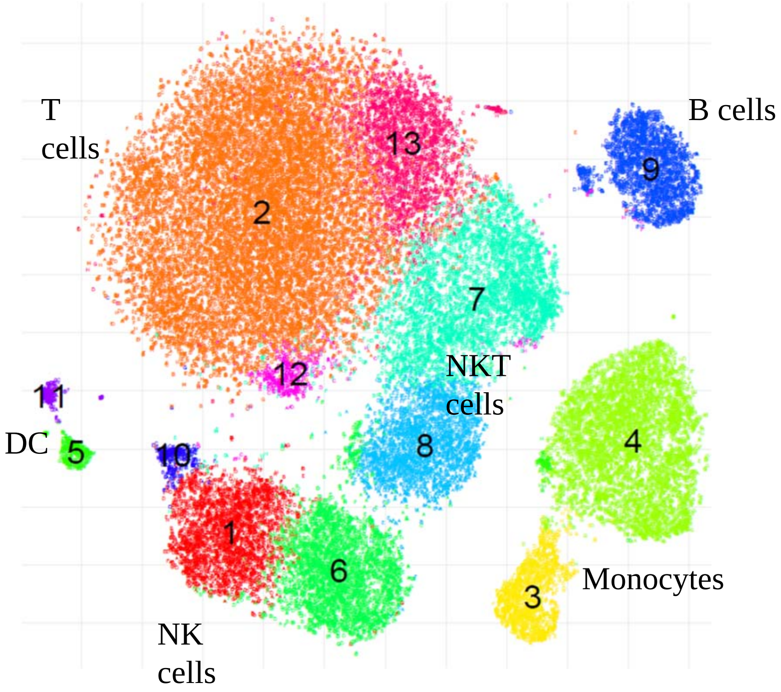

B

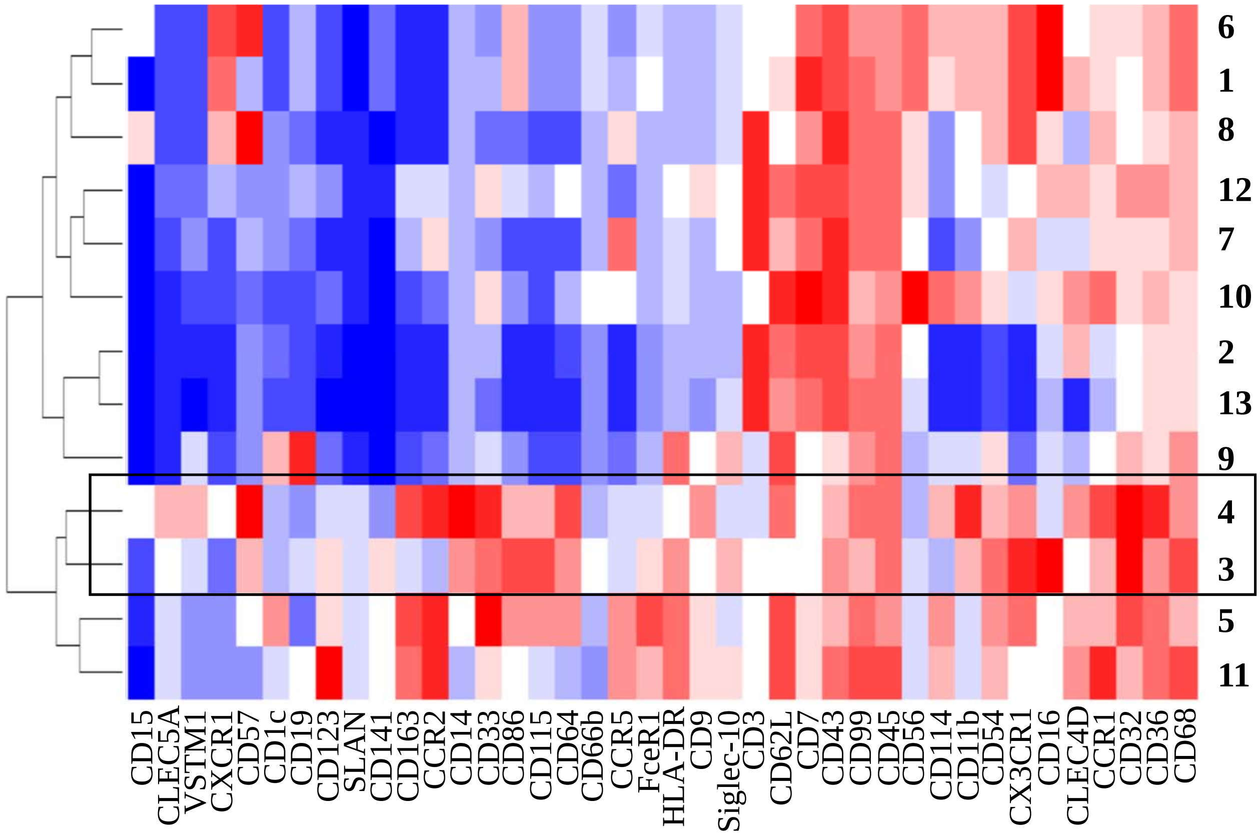

Supp Figure S1. Visualization of the expression of forty markers on PBMCs. (A) *t*-SNE clustering of PBMCs based on the expression of the forty markers. **(B)** Expression level of the forty markers on each cluster in (A).

Supplementary Figure S2

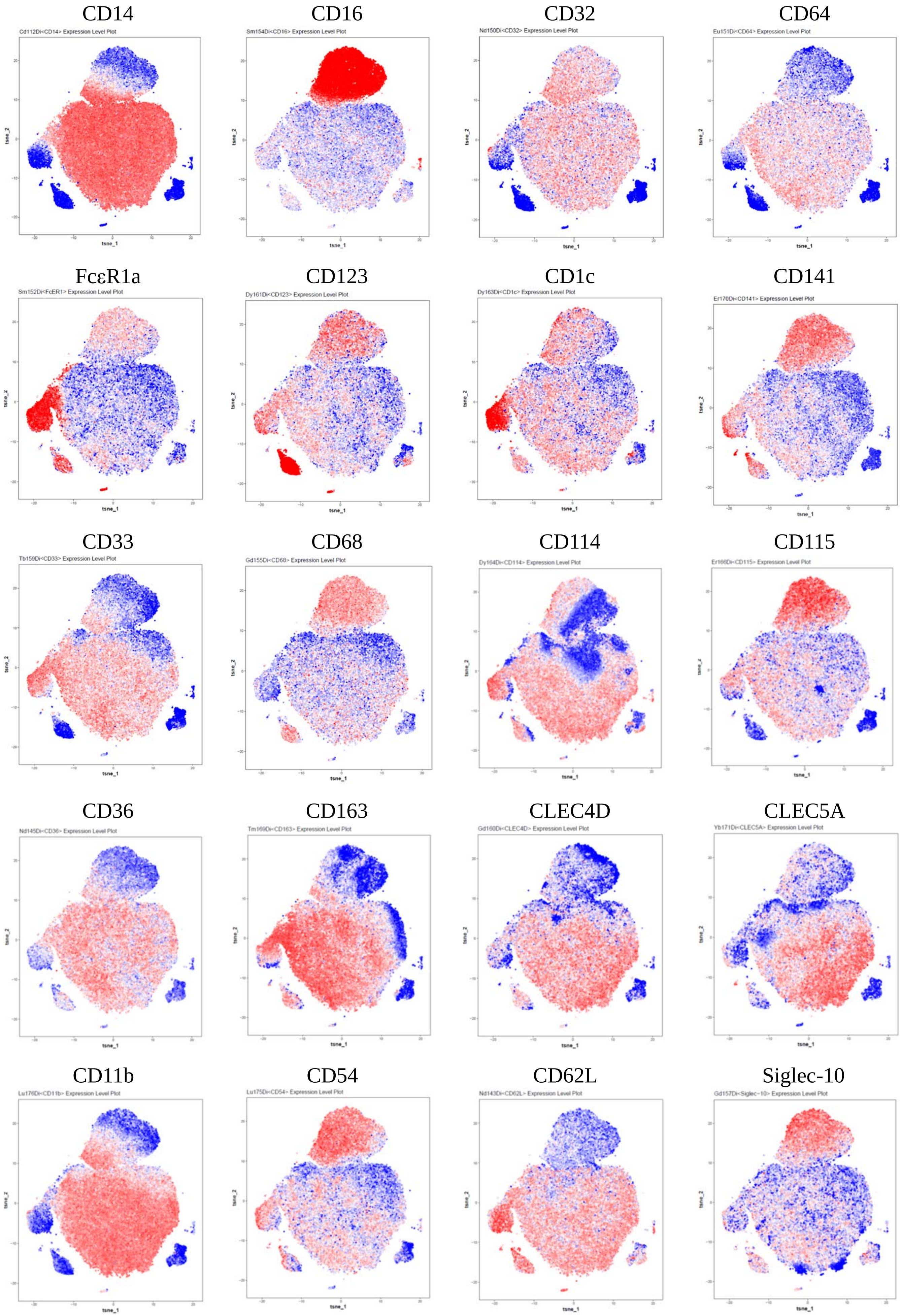

Supplementary Figure S2 (continued)

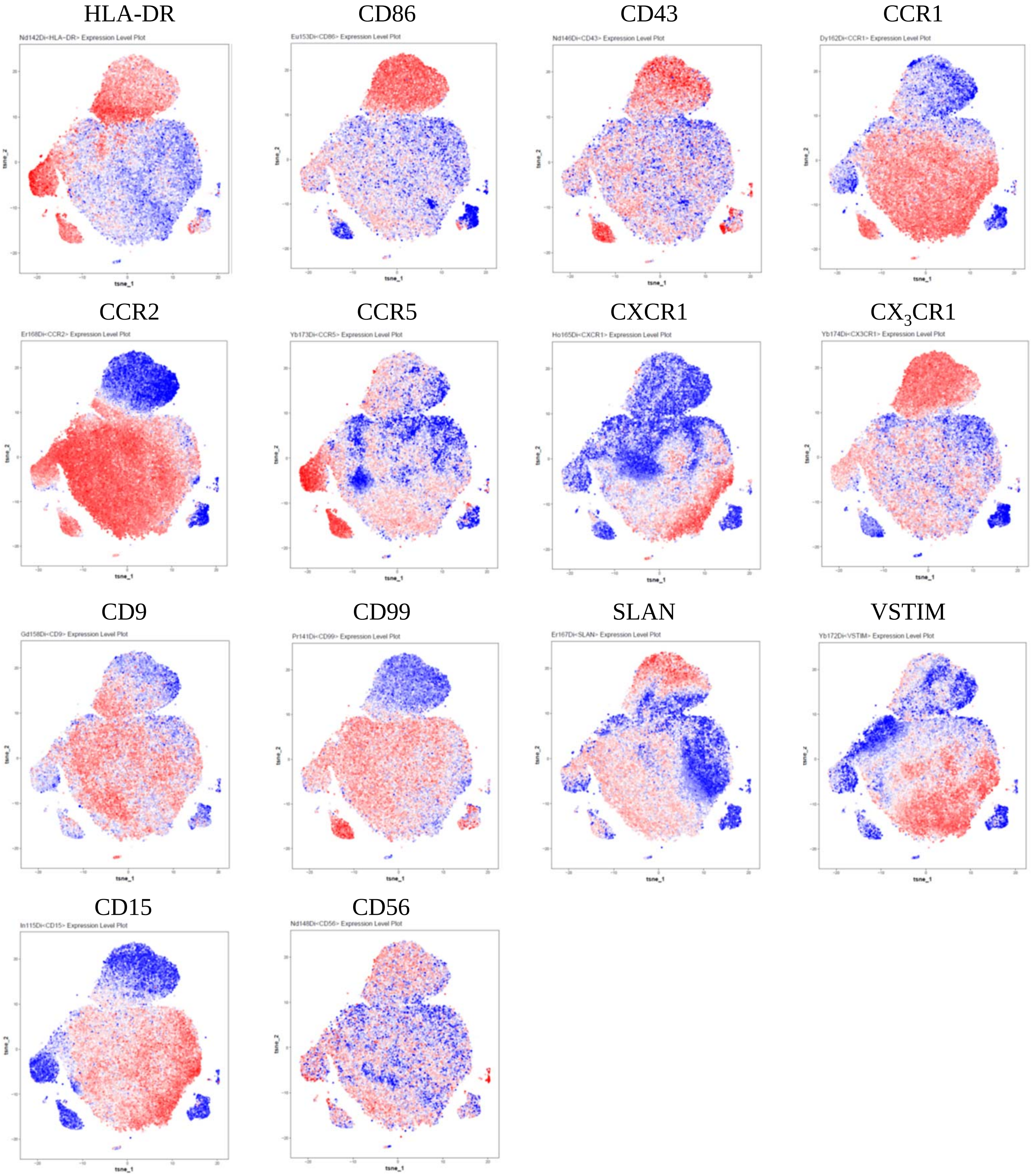

Supp Figure S2. Expression level of each of the thirty-four markers on monocytes visualized on *t*-SNE plots.

Supplementary Figure S3

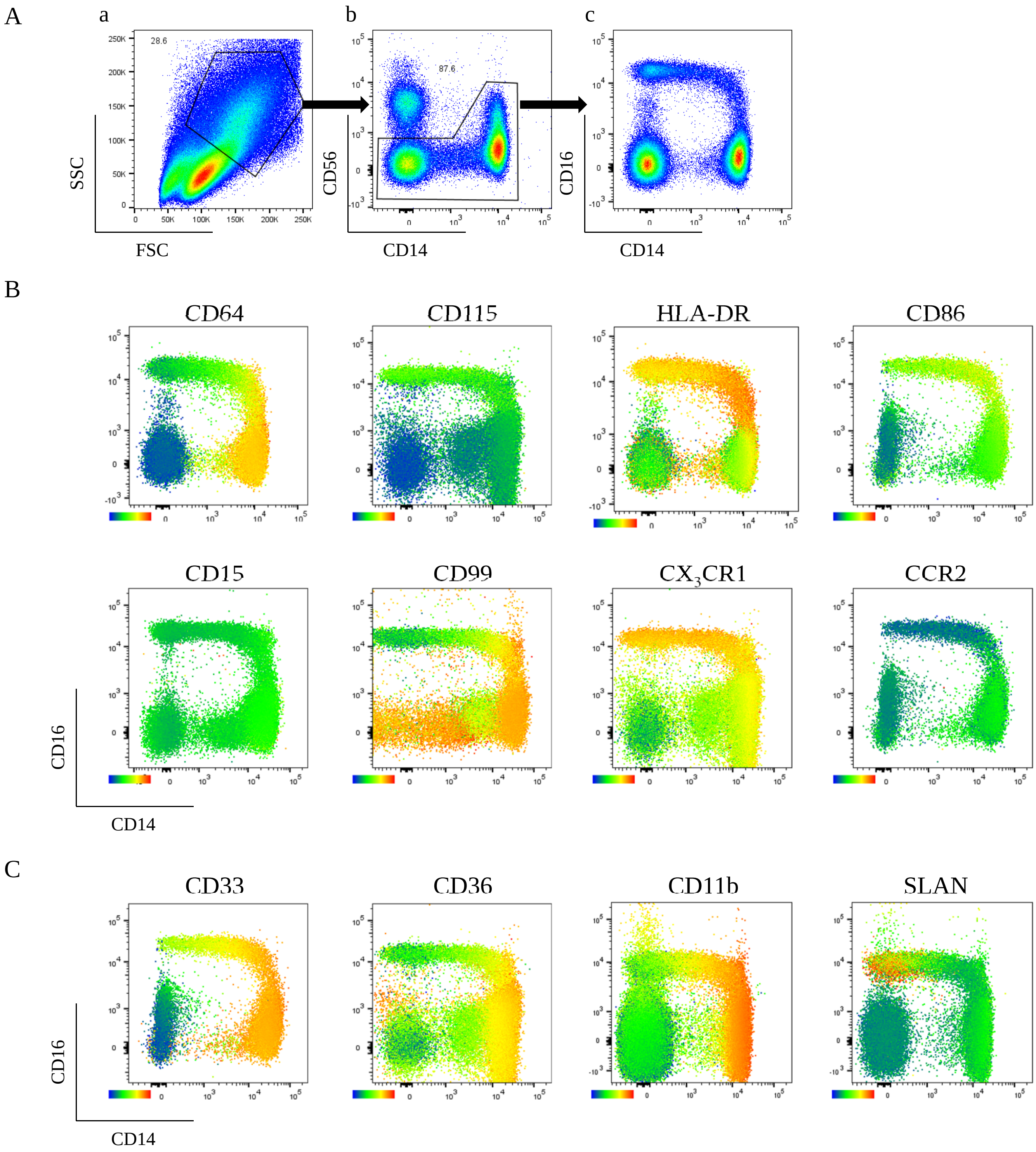

Supp Figure S3. Markers shortlisted from CyTOF data were assessed with flow cytometry on a conventional CD16-CD14 plot. (A) Enriched monocytes were gated from PBMCs for (B) and (C). Heatmap expression of markers shortlisted to separate $CD16^{+}$ and $CD16^{-}$ monocytes **(B)** and NC and ITM subsets **(C)**.

Supplementary Figure S4

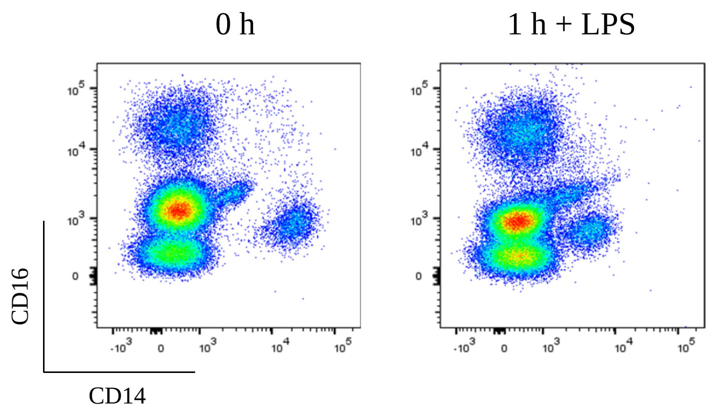

Supp Figure S4. Down-regulation of CD14 and CD16 on monocytes in whole blood after 1 h of stimulation with LPS.

Supplementary Figure S5

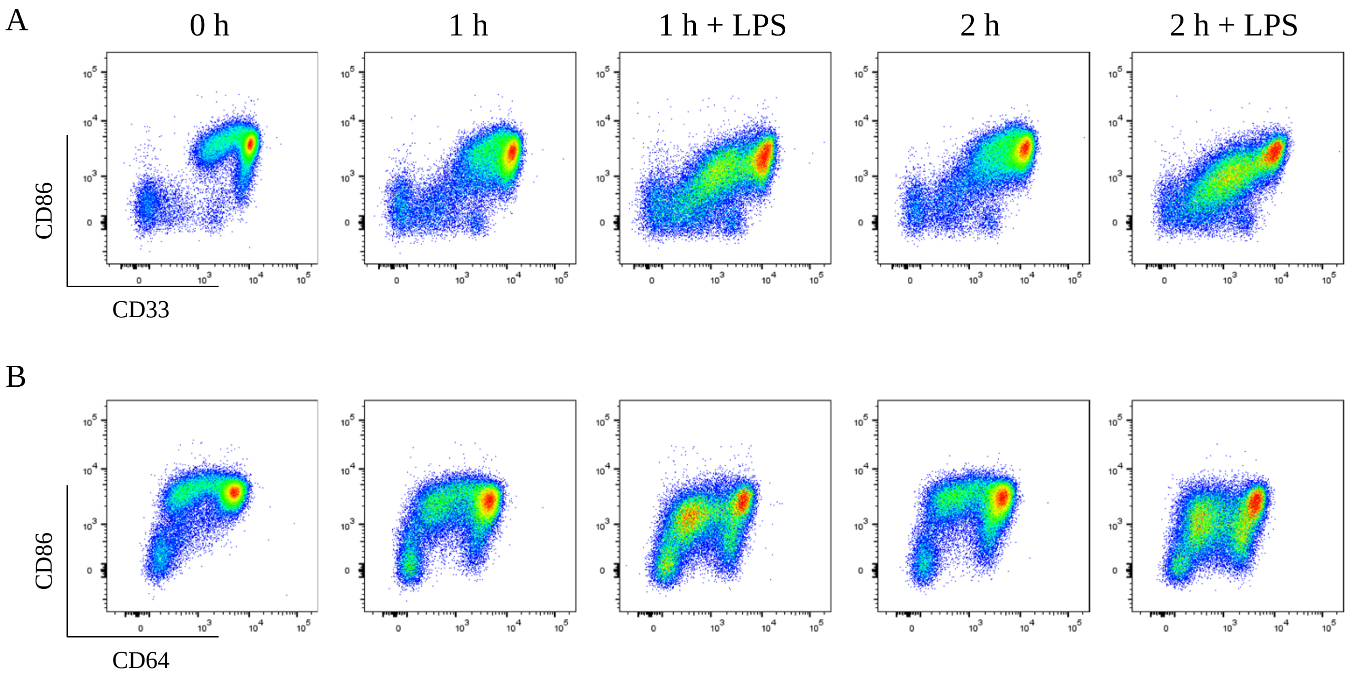

Supp Figure S5. Expression of CD33 on monocytes **(A)** is more modulated than that of CD64 **(B)** *in vitro*.
